# Cell viability assessed in a reproducible model of human osteoblasts derived from human adipose-derived stem cells

**DOI:** 10.1371/journal.pone.0194847

**Published:** 2018-04-11

**Authors:** Regiane M. C. Olimpio, Miriane de Oliveira, Maria T. De Sibio, Fernanda C. F. Moretto, Igor C. Deprá, Lucas S. Mathias, Bianca M. Gonçalves, Bruna M. Rodrigues, Helena P. Tilli, Virgínia E. Coscrato, Sarah M. B. Costa, Gláucia M. F. S. Mazeto, Célio J. C. Fernandes, Willian F. Zambuzzi, Patrícia P. Saraiva, Durvanei A. Maria, Célia R. Nogueira

**Affiliations:** 1 Department of Internal Medicine, Botucatu Medical School, São Paulo State University - UNESP, Botucatu, São Paulo, Brazil; 2 Institute of Biosciences, Department of Chemistry and Biochemistry, São Paulo State University - UNESP, Botucatu, São Paulo, Brazil; 3 Biochemistry and Biophysics Laboratory, Butantan Institute, São Paulo, São Paulo, Brazil; Università degli Studi della Campania, ITALY

## Abstract

Human adipose tissue-derived stem cells (hASCs) have been subjected to extensive investigation because of their self-renewal properties and potential to restore damaged tissues. In the literature, there are several protocols for differentiating hASCs into osteoblasts, but there is no report on the control of cell viability during this process. In this study, we used osteoblasts derived from hASCs of patients undergoing abdominoplasty. The cells were observed at the beginning and end of bone matrix formation, and the expression of proteins involved in this process, including alkaline phosphatase and osteocalcin, was assessed. RANKL, Osterix, Runx2, Collagen3A1, Osteopontin and BSP expression levels were analyzed using real-time PCR, in addition to a quantitative assessment of protein levels of the markers CD45, CD105, STRO-1, and Nanog, using immunofluorescence. Rhodamine (Rho123), cytochrome-c, caspase-3, P-27, cyclin D1, and autophagy cell markers were analyzed by flow cytometry to demonstrate potential cellular activity and the absence of apoptotic and tumor cell processes before and after cell differentiation. The formation of bone matrix, along with calcium nodules, was observed after 16 days of osteoinduction. The gene expression levels of RANKL, Osterix, Runx2, Collagen3A1, Osteopontin, BSP and alkaline phosphatase activity were also elevated after 16 days of osteoinduction, whereas the level of osteocalcin was higher after 21 days of osteoinduction. Our data also showed that the cells had a high mitochondrial membrane potential and a low expression of apoptotic and tumor markers, both before and after differentiation. Cells were viable after the different phases of differentiation. This proposed methodology, using markers to evaluate cell viability, is therefore successful in assessing different phases of stem cell isolation and differentiation.

## Introduction

The use of adipose tissue-derived stem cells (ASCs) has contributed to clinical and experimental research in a variety of biological systems [1]. Similar to bone marrow, adipose tissue is derived from the mesodermal germ layer and contains a supportive stroma that contains ASCs, which can be easily separated away from adipose cells [2]. Furthermore, it has been well documented that fat is an endocrine organ that releases numerous hormones, referred to as adipokines, that are involved in the control of body physiology, and are also important for the sustained maintenance of healthy ASCs [3]. Human ASCs (hASCs) have been studied extensively because of their self-renewal capability and their potential to restore damaged tissues that have reduced self-regenerative capabilities, such as cartilage, bone [4–6] hASCs can also differentiate into different lineages, such as adipogenic, chondrogenic, and myogenic [7].

The hASCs are characterized by their expression of mesenchymal (CD90 and CD105) [8–10] and pluripotency (Nanog, Sox2, and Stro-1) markers [11]. However, this cell type does not express the hematopoietic markers, CD45 [9, 11] CD34, and CD117 [11]. hASCs can differentiate into osteoblasts [12] when cultured in an appropriate osteogenic differentiation media [13]. This property makes hASCs a useful experimental model that allows for an understanding of the behavior of osteoblasts during the different stages of osteoinduction [4].

Although there are several studies in the literature that report different protocols for the differentiation process, we have also identified the need to control cell viability. The process of hASC differentiation into osteoblasts involves several steps, during which the hASCs can become damaged, so assessing ASC viability during the osteoblastic process is important if these cells are to be useful experimentally.

Hence, the present study describes the use of various markers to assess cell viability during the differentiation, starting from the isolation of stem cells from adipose tissue to their subsequent differentiation into osteoblasts.

## Materials and methods

### hASC isolation

The Ethics Committee of the Botucatu Medical School, São Paulo State University (UNESP) approved this study under number protocol 3216–2009. The hASCs were isolated from subcutaneous adipose tissue obtained from six patients undergoing abdominoplasty after weight loss induced by bariatric surgery, at the Plastic Surgery Department of the Botucatu Medical School. Abdominoplasty patients up to 50 years of age with normal erythrocyte sedimentation rate (ESR) were included in the study. All patients included in this study provided written informed consent. Subcutaneous adipose tissue samples were then submitted to enzymatic digestion. Briefly, 2 g of adipose tissue was incubated with 4 mg type I collagenase in 8 mL of phosphate-buffered saline (PBS). Initially, the isolated hASCs were plated at a density of 2 × 10^5^ in a T25 flask, and grown in a complete medium, defined as Dulbecco's modified Eagle medium (DMEM), containing 10% fetal bovine serum (FBS) with 1% penicillin-streptomycin and 0.1% gentamicin (10 mg/mL; Invitrogen). Upon reaching 70% confluency, cells were trypsinized and transferred to a T75 flask for cell expansion. All cell cultures were maintained at 37°C in a humidified atmosphere of 95% O_2_ and 5% CO_2_. Aliquots of hASCs were prepared at the second passage and frozen in liquid nitrogen until cell characterization.

### hASC characterization by flow cytometry

Fixation and permeabilization methods were for the detection of intracellular antigens by flow cytometry of the hASCs. The detection of intracellular antigens requires a cell permeabilization step prior to staining. The cells are fixed with paraformaldehyde to stabilize the cell membrane, and then permeabilized with 5 μl of Triton X-100 (0.1%) for 30 min, prior to incubation with primary antibodies specific to allow for permeation of antibodies against intracellular antigens.

The cells were then stained with a range of different antibodies at a concentration of 1 mg/mL each at 4°C for 30 min. The antibodies used for analysis were CD105 (AbD Serotec, Raleigh, NC, USA), CD45RO (Sigma-Aldrich St. Louis, MO, USA), CD90, CD117, Nanog, STRO-1, and Sox-2 (Abcam Cambridge, MA, USA). The corresponding isotype antibody was used as a negative control, and the secondary antibody used was goat anti-mouse IgG (H/L)-FITC (AbD Serotec).

Cell fluorescence was analyzed on a FACS Calibur flow cytometer (Becton Dickinson, San Jose, CA, USA) using Cell Quest 2.9 software and Vit MDI, which were employed for acquisition and histogram analysis. Cell auto-fluorescence was measured using flow cytometry, using cells incubated without fluorochrome-labeled antibodies. The fluorescence intensity was measured for the positive and negative markers, FL1 and FL2, at wavelengths generated by the excitation of the fluorochromes with light at the maximal wavelength. The expression of the different surface antigens was determined using the antibodies described above, and labeling was performed according to the supplier’s recommendations.

### Vimentin staining in hASCs

First, hASCs were cultured on round cover slips (12 × 0.11 mm). Following this, the samples were fixed using acetone for 10 min, and thereafter washed with ice-cold PBS and incubated for 30 min in 1% N,O-Bis trimethylsilyl acetamide (BSA) solution. After this period, the cells were incubated with the specific antibodies for 1 hour. To label hASCs, an anti-vimentin antibody was used (mouse monoclonal, clone Vim 3B4, Produktionsvej/Dako Denmark). After 1 hour, the cells were washed again using PBS and incubated with a secondary antibody NHS DyLight 350 (Thermo Fisher Scientific, Waltham, MA, USA) for 1 hour. Lastly, the cells were incubated for 1 min with DAPI for nuclear staining, and immunofluorescence was visualized using an Olympus microscope (UTV063XC/SNJ41986).

### hASC differentiation and osteoblast culture

Freshly-isolated hASCs were cultured in DMEM containing 10% FBS with 1% penicillin-streptomycin (10,000 g/mL-10,000 μ/mL, Invitrogen) and 0.1% gentamicin (10 mg/mL; Invitrogen). For hASC differentiation into osteoblasts, complete DMEM medium supplemented with 0.1 μM dexamethasone (Sigma-Aldrich), 50 μM ascorbic acid (Sigma-Aldrich), and 10 mM β-glycerophosphate was used (Sigma-Aldrich). Cells were maintained in this medium for a period of 7, 16, or 21 days. The culture medium was replaced three times per week with fresh medium containing the same osteogenic components. hASCs were used as a negative control for osteogenic induction, and were cultured in DMEM in the absence of osteogenic compounds. Coinciding with the exchange of culture medium, cells were visually examined using an inverted microscope, and cell images were obtained using a digital camera.

### Formation of mineralized matrix of the osteoblasts

The cell monolayer present in the petri dish was washed with PBS after which 1X paraformaldehyde was used to fix the cells (20 min). After removal of the paraformaldehyde, the cells were washed with distilled water. In order to visualize the mineralized matrix, cells were stained with Alizarin Red for 5 minutes at a temperature of 26°C. Alizarin Red staining was performed after incubation in 10% cetylpyridinium chloride and 10 mM sodium phosphate (pH 7.0) for 15 min. The concentration of Alizarin Red was determined by measuring the absorbance at 562 nm.

### Alkaline phosphatase (ALP) and osteocalcin assay

After 7, 16 and 21 days of osteoinduction, 2 mL of the culture medium were collected for the alkaline phosphatase and osteocalcin assays. A colorimetric assay was used for the determination of ALP activity (calcium kit, Sigma and Procedure n° 0150, Stanbio Laboratory) and osteocalcin, the Osteocalcin ELISA kit (Takara BioInc., Shiga, Japan) according to the instructions of the manufacturer [14]. ALP staining on hASC and osteoblasts (16 day of osteoinduction), was done with FAST BCIP/NBT (Sigma-Aldrich, St. Louis, Missouri, USA) (Catalog number B5655), after fixing the cells with methanol according to the manufacturer´s instructions. Sigma FAST BCIP/NBT active substrate solution containing BCIP (0.15 mg/ml), NBT (0.30 mg/ml), Tris buffer (100 mM), and MgCl2 (5 mM), pH 9.25–9.75 was prepared by dissolving one tablet in 10ml of water. The BCIP-NBT substrate is hydrolyzed/reduced by ALP, when osteoblast positive, to form a deep-purple/black precipitate which is readily detectable by eye and was photographed in Olympus microscope (U-TV0.63XC).

### Gene expression of RANKL, Osterix, Runx2, Collagen3A1, Osteopontin and BSP on osteoblasts

Total mRNA was extracted from osteoblasts using the Trizol method (Invitrogen) according to the manufacturer's instructions. A High Capacity cDNA Reverse Transcription Kit for Real-Time PCR (RT-PCR)^®^ (Invitrogen, São Paulo, Brazil) was used for the synthesis of 20 μL of complementary DNA (cDNA) from 1,000 ng total RNA. The expression levels of BSP (forward = GTTGCGTCTTGGAAGTGAGA; reverse = CAGCTGGCTGATCACTCAAA), Collagen3A1 (forward = AACCAAGGCTGCAACCTGGA; reverse = GGCTGAGTAGGGTACACGCAGG), Runx2 (forward = CCGTCCATCCACTCTACCAC; reverse = ATGAAATGCTTGGGAACTGC), Osterix (forward = CCAGGCAACACTCCTACTCC; reverse = GCCTTGCCATACACCTTGC), Osteopontin (forward = ATGAGAGCCCTCACACTCCTC; reverse GCCGTAGAAGCGCCGATAGGC) and GAPDH (forward = GACTCATGACCACAGTCCATGC; reverse = AGAGGCAGGGATGATGTTCTG) were analyzed by real-time quantitative PCR (RT-qPCR), reaction condicitions where 95°C- 3s; 60°C-8s; 72°C-20s. The expression levels of RANKL (Applied Biosystems TNFRSF11-Hs00243522_m1 assay), was performed using the TaqMan commercial qPCR kit (Invitrogen) according to the manufacturer's instructions. The amplification conditions were as follows: enzyme activation at 50°C for 2 min, denaturation at 95°C for 10 min, amplification of the cDNA products by 40 cycles of denaturation at 95°C for 15 s, and annealing/extension at 60°C for 1 min. After normalization to the internal control GAPDH (Applied Biosystems 02758991_g1 test Hs) (19) by the 2^-ΔΔCt^ method, as previously described [15], RANKL mRNA expression was determined in control hASCs and osteoblasts after 7, 16, and 21 days. The other markers, Osterix, Runx2, Collagen3A1, Osteopontin and BSP were determined hASCs and osteoblasts after 16 days with osteoinduction medium.

### Fluorescence microscopy for osteoblast markers

hASCs were plated on glass coverslips and differentiated into osteoblasts. After 16 days, the osteoblasts were fixed using 4% formaldehyde. Subsequently, the cover slips were washed and incubated in 50 nM NH_4_Cl with PBS. After this period, cells were washed three times with PBS and permeabilized with 0.1% saponin in PBS. Cells were incubated with the appropriate primary antibody, followed by incubation with a DyLight 488-conjugated secondary antibody (1:200), and mounted with media containing DAPI. The antibodies used were anti-RANKL, anti-Stro1, anti-Nanog, anti-CD105, and anti-CD45RO. Osteoblasts were analyzed using confocal microscopy and photographed and immunofluorescence was visualized using an Olympus microscope (U-TV0.63XC). Fluorescence was measured for each protein using NIS Elements AR 3.2 software.

### Evaluation of cell viability of hASC and osteoblasts

The mitochondrial membrane potential was measured using a rhodamine (Rho123) based assay, and monitored by flow cytometry. hASC and osteoblast were incubated in a Rho 123 solution (100 mg/mL) diluted in DMSO (5 μg/mL) in a 5% CO_2_ incubator for 30 min.

After washing with PBS, the cells were analyzed using a Guava^®^easyCyte Flow Cytometer. A total of 10000 cells/sample were analyzed and the mean fluorescence intensity and percentage of cells in each population was recorded.

For the expression of cytochrome-c, caspase 3, P27, cyclin D1 markers, and for the evaluation of autophagy (p62), the cells were first fixed with 1% paraformaldehyde and then permeabilized with 0.02% Triton X-100, then stained with anti-cytochrome c, anti caspase 3, anti P27, anti-and cyclin D1 and anti p62 antibodies, or isotype control antibody (Santa Cruz Biotechnology, Santa Cruz, CA, USA). The percentages of cells expressing the respective markers were quantified by flow cytometry (Guava^®^easyCyte Flow). A total of 10,000 cells per sample was analyzed and the mean fluorescence intensity and percentage of cells in each population were determined.

### Statistical analysis

All data were evaluated by the D'Agostino & Pearson normality test. Analysis of variance (ANOVA) was performed followed by a Tukey test. Student's t test was used for comparison between two experimental groups. Data are expressed as mean ± standard deviation. The level of significance was set at value p < 0.05.

## Results

### Morphology of hASCs

**Fig 1:**
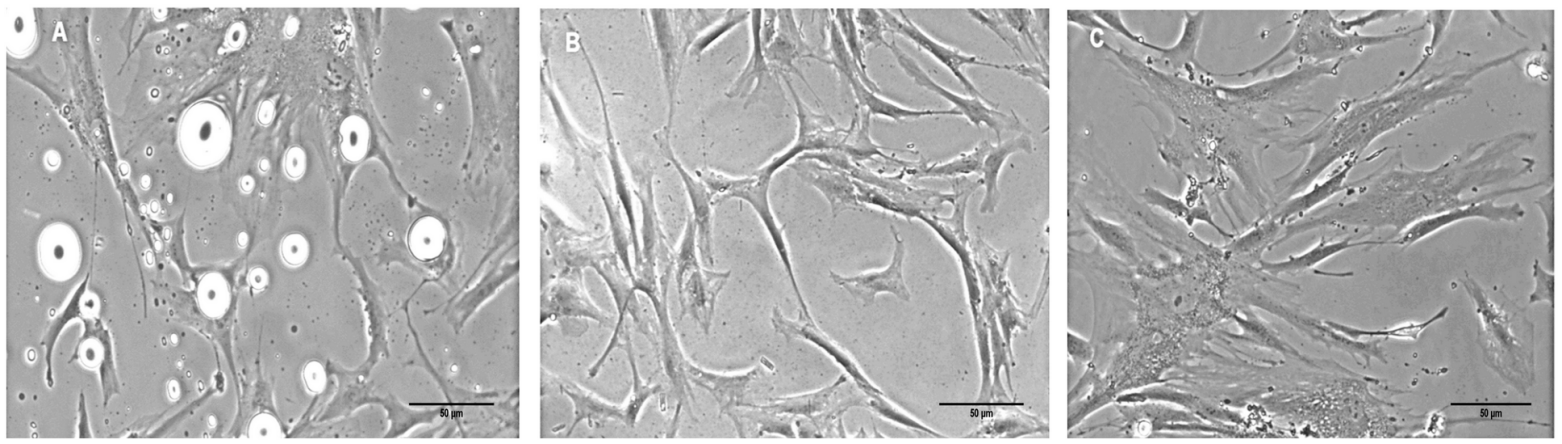
Morphology of hASCs. (A) hASCs containing fat droplets adhered at the first passage after isolation. (B, C) hASCs at the second and third passages, respectively. In all the passages observed, the cells show adhesion to the plastic and had a morphology similar to fibroblasts.

After the first passage, hASCs clearly showed the presence of fat droplets (Fig 1A), which disappeared after the second and third passages (Fig 1B and 1C, respectively). hASCs exhibited a fibroblastoid morphology a few days after being isolated from the adipose tissue, and this morphology was maintained during the later passages.

### Expression of markers in hASCs

**Fig 2:**
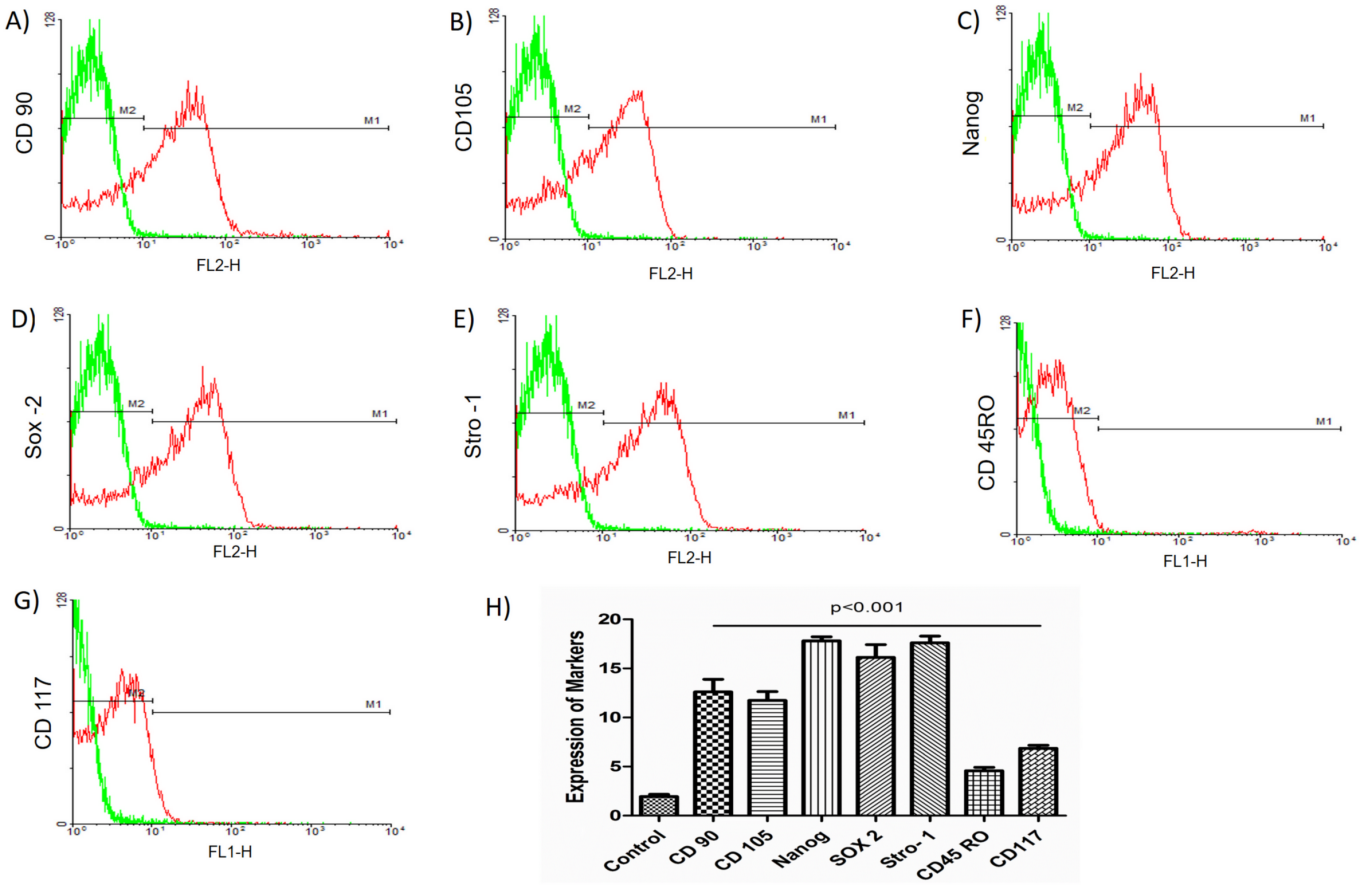
Analysis of hASC markers. (A) CD90. (B) CD105. (C) Nanog. (D) Sox-2. (E) STRO-1. (F) CD45RO. (G) CD117. (H) Quantification of expression of positive and negative markers. Graphs show the expression of different markers in hASCs. The expression of the markers was assessed using flow cytometry by means of the intensity of the emitted fluorescence (FL1 and FL2). The expression of the positive markers, CD90, CD105, and STRO-1 (surface markers mesenchymal) and Nanog and Sox-2 (pluripotency markers) is compared to the decreased expression of negative markers (CD45RO and CD117). Data are represented as mean ± standard deviation. The statistical analyses were obtained using an ANOVA followed by Dunnett’s test with P < 0.05 being considered significant.

The expression levels of the positive (CD90, CD105, Nanog, Sox-2, and STRO-1) and the decrease in the expression of the negative markers (CD45RO and CD117) were analyzed in hASCs by flow cytometry as shown in Fig 2. The expressions of Nanog and Sox-2 in hASCs demonstrate that the cells remained undifferentiated, and thus indicates the conservation of pluripotency in these cells. All positive markers had significantly higher levels of expression levels than the negative markers (P < 0.001).

### Presence of vimentin in hASCs

**Fig 3:**
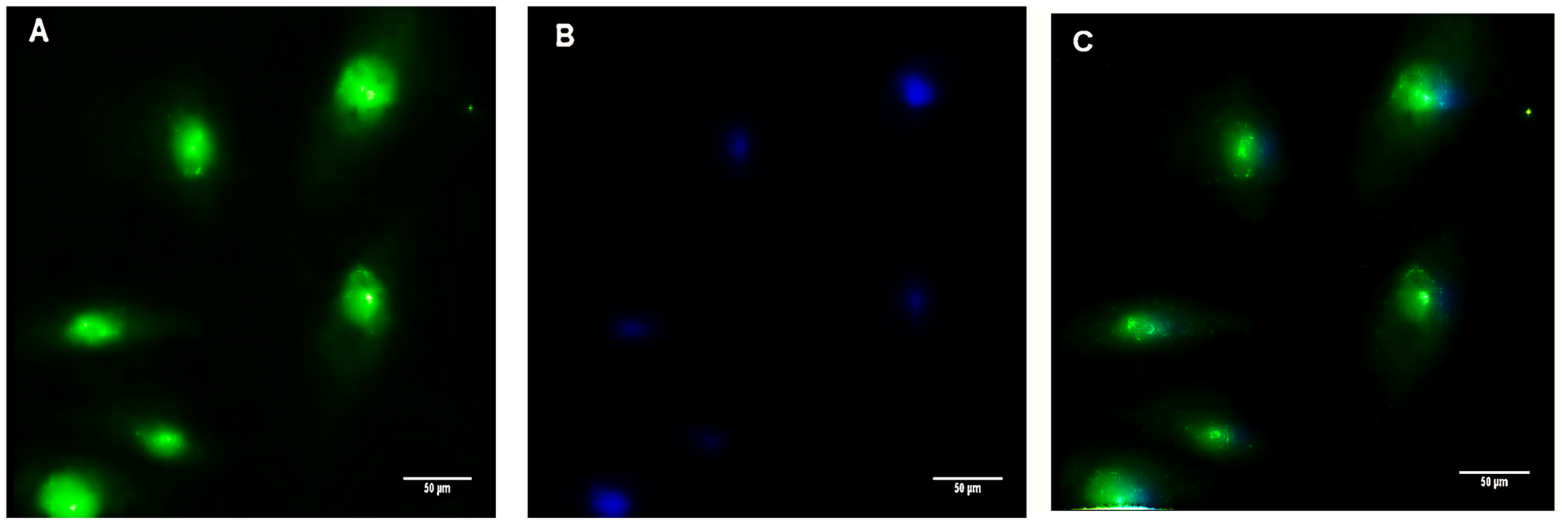
Presence of vimentin in hASCs. (A) Vimentin expression is often used as a marker for cells and tissues derived from the mesoderm. The presence of this protein confirmed the mesenchymal origin of the cells. (B) Image of nuclear staining assessed using DAPI staining. (C) Merged images.

Fig 3 shows the staining for vimentin, a mesenchymal marker, along with DAPI staining for nuclei. Cytoplasmic labeling of vimentin in hASCs revealed that this protein was present in mesodermal cells that had originated from stem cells.

### Mineralized matrix at different days of osteoinduction

**Fig 4:**
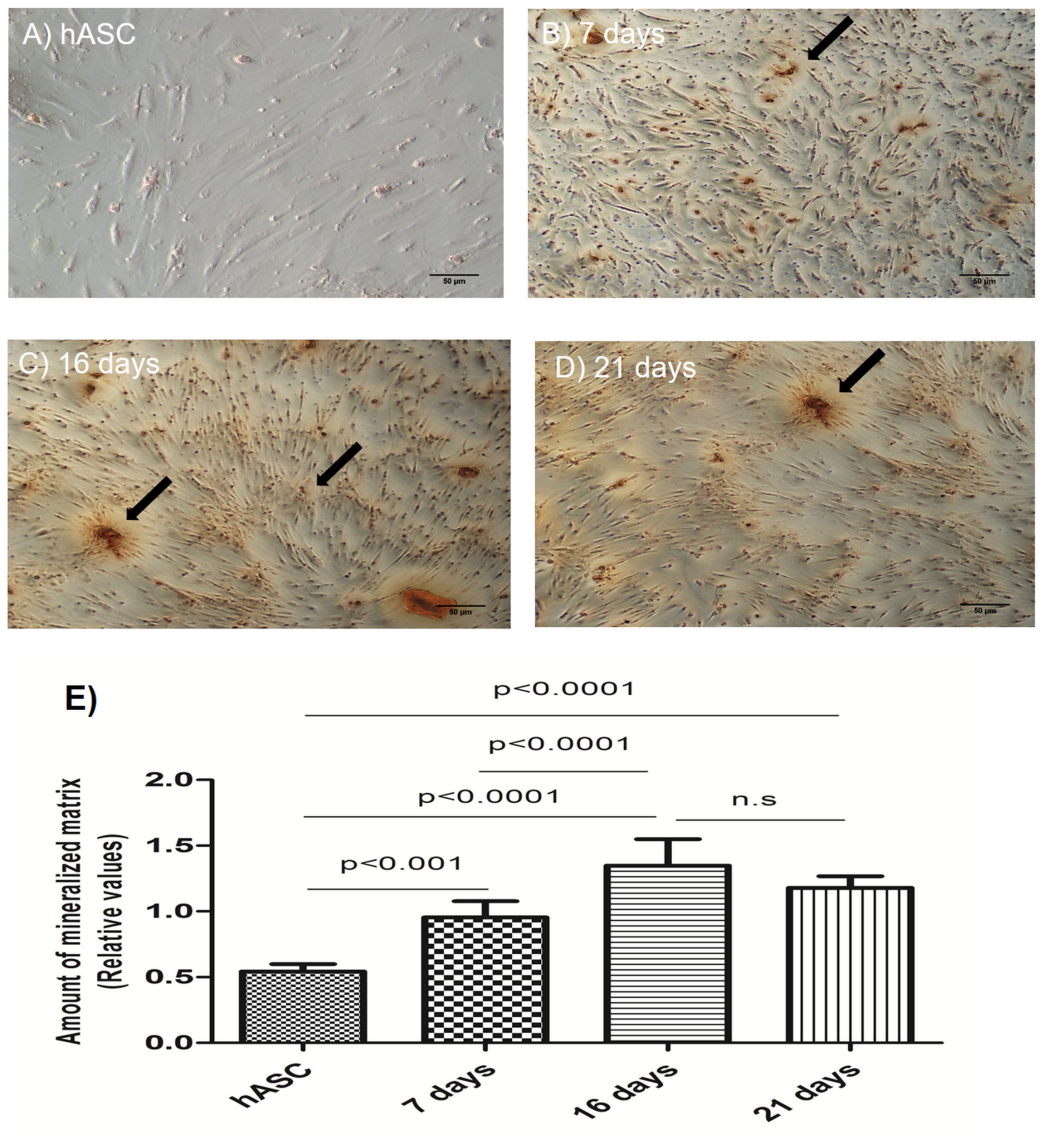
Presence of mineralized matrix in mature osteoblasts. (A) hASCs used as the negative controls. (B) Image of cells cultured for 7 days, showing a less intense staining. (C) Images of cells cultured for 16 days showing a more intense staining, and the presence of a mineralized matrix with the accumulation of calcium nodules being clearly visible (indicated by the arrows). (D) The matrix and calcium nodules remained visible 21 days post-osteoinduction. (E) The amount of mineralized matrix on different days of osteoinduction. n.s: non-significant. Data are expressed as mean ± standard deviation. An ANOVA followed by Tukey’s test was used to analyze the data. P < 0.005.

The amount of mineralized matrix observed in all periods of osteoinduction was larger than in hASCs. At 16 and 21 days there was a greater formation of mineralized matrix that at 7 days (Fig 4). There was no statistical difference between the amount of mineralized matrix at 16 and 21 days.

### Gradual increase in alkaline phosphatase and osteocalcin post-osteoinduction

**Fig 5:**
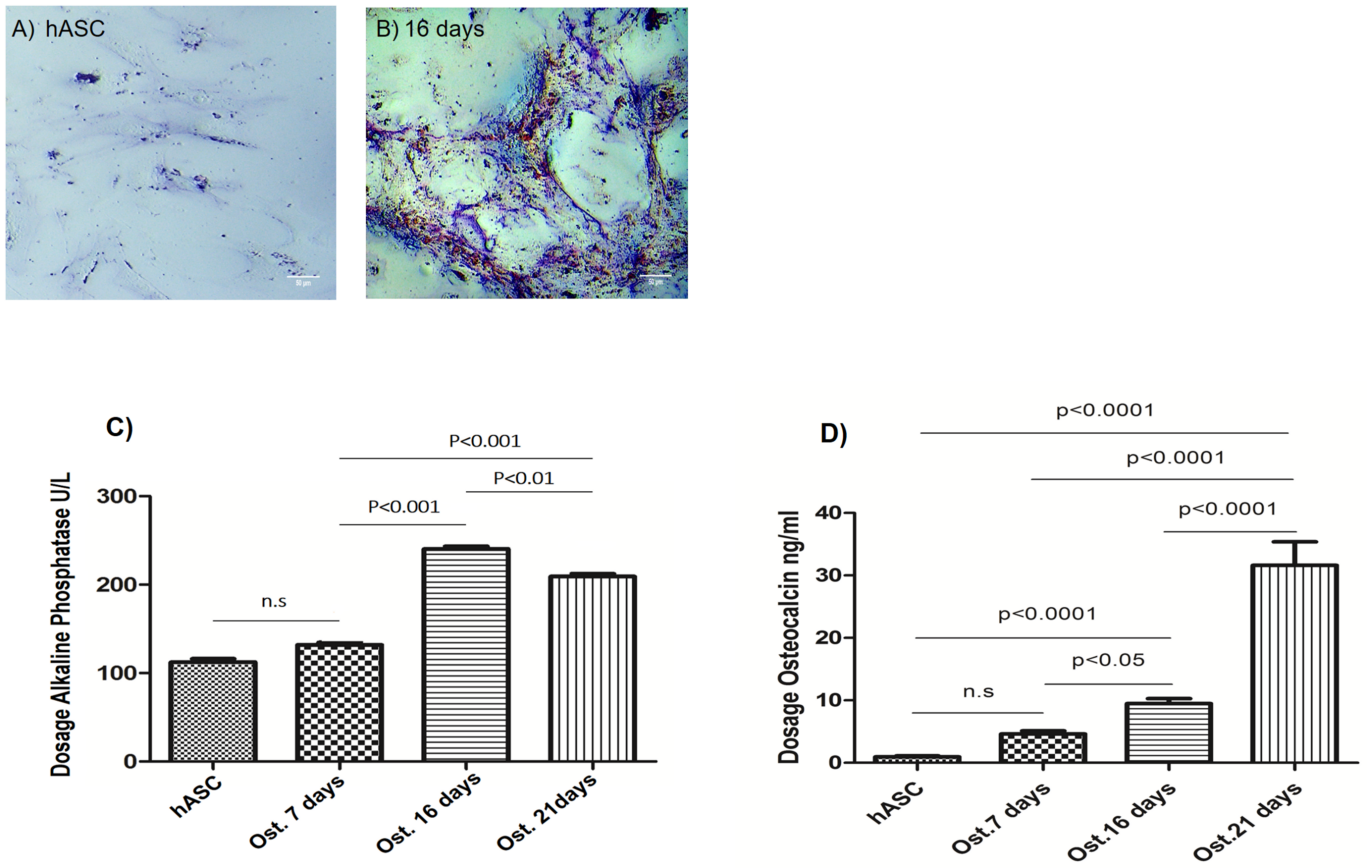
Gradual increase in alkaline phosphatase and osteocalcin post-osteoinduction. (A) Alkaline phosphatase staining in hASC. (B) Alkaline phosphatase staining in osteoblasts cultured for 16 days with osteoinduction medium. (C) Level of alkaline phosphatase. (D) Level of osteocalcin. Mesenchymal stem cells derived from human adipose tissue (hASCs). Osteoblasts cultured for 7 days with osteoinduction medium (Ost. 7 days). Osteoblasts cultured for 16 days with osteoinduction medium (Ost. 16 days). Osteoblasts cultured for 21 days with osteoinduction medium (Ost. 21 days). n.s: non-significant. Data are expressed as mean ± standard deviation. An ANOVA followed by Tukey’s test was used to analyze the data. P < 0.005.

We demonstrated the presence of the more intense alkaline phosphatase in osteoblasts culture medium at 16 days post osteoinduction in relation to hASC (Fig 5C) and is evidenced by the staining of the ALP in osteoblast (Fig 5B) in relation as hASC (Fig 5A). The Fig 5D show a quantification of osteocalcin levels in the culture medium at 7, 16, or 21 days post-osteoinduction. The activity of both of these proteins was confirmed in osteoblasts. There was a gradual increase in the expression of both proteins between 7 and 16 days after the start of differentiation. In the early stages of differentiation, the levels of these enzymes contribute to the onset of bone mineralization.

### RANKL, Osterix, Runx2, Collagen3A1, Osteocalcin and BSP mRNA expression in osteoblasts

**Fig 6:**
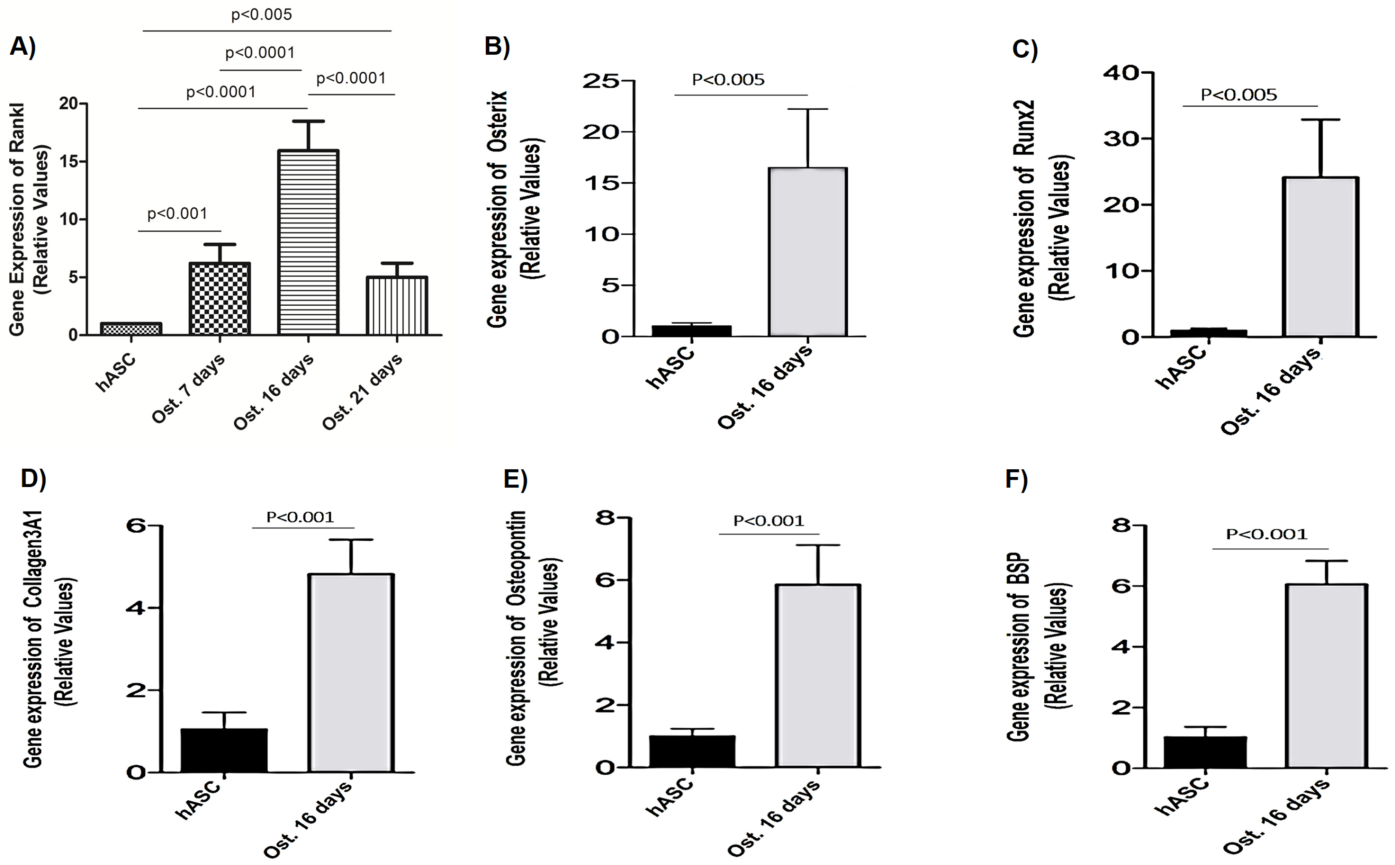
Gene expression of RANKL, OSTERIX, RUNX2, COLLAGEN3A1, OSTEOPONTIN and BSP after varying times of osteoinduction. (A) Cells were cultured in an osteogenic medium for 7, 16, or 21 days to RANKL gene. RANKL genes expression was the highest at 16 days post osteoinduction as well as the osteogenic markers Osterix, Runx2, Collagen3A1, Osteopontin and BSP (Fig 6B-6F). Mesenchymal stem cells derived from human adipose tissue (hASCs). Osteoblasts cultured for 7 days with osteoinduction medium (Ost. 7 days). Osteoblasts cultured for 16 days with osteoinduction medium (Ost. 16 days). Osteoblasts cultured for 21 days with osteoinduction medium (Ost. 21 days). An ANOVA followed by Tukey's test was used to analyze the data. P <0.005.

RANKL, OSTERIX, RUNX2, COLLAGEN3A1, OSTEOCALCIN and BSP mRNA expression were evident in mature osteoblasts derived from hASCs after 16 days of differentiation (Fig 6A–6F). These results indicate that in this period of osteoinduction, these proteins are highly involved in the processes of mineralization and bone formation. There was a decrease in the expression of RANKL mRNA by 21 days, the levels being similar to those observed at 7 days (Fig 6A); this justifies measure the other markers only in the 16 day period.

### Evaluation of mature osteoblast markers

**Fig 7:**
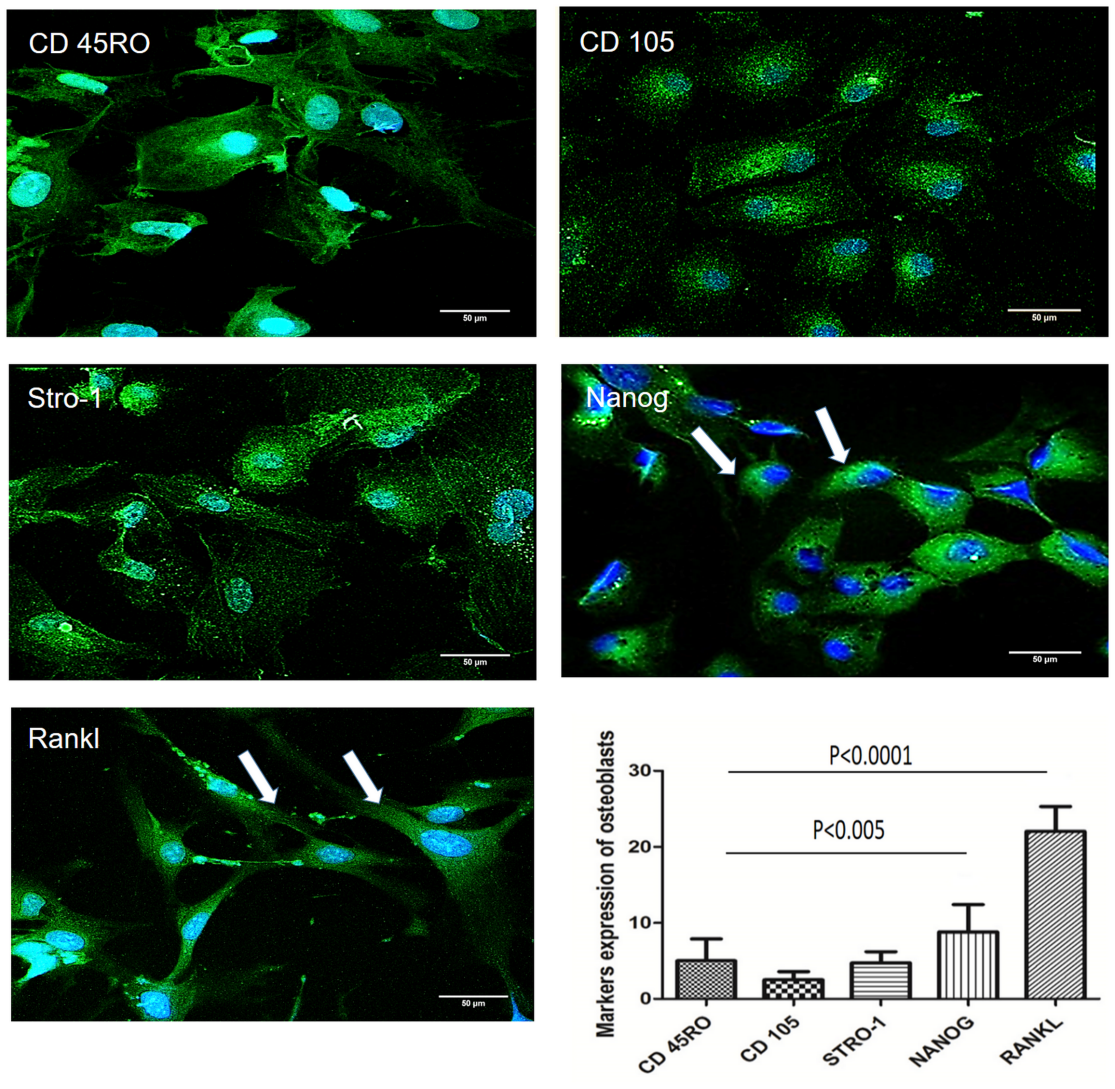
Analysis of the markers present in osteoblasts. (A) CD45RO. (B) CD105. (C) STRO-1. (D) The arrows indicate the perinuclear location of Nanog. (E) The arrows indicate the location of RANKL. (F) Levels of expression of markers. Cell images showing the expression of the markers CD 45RO, RANKL, Nanog, CD105, and STRO-1 in osteoblasts cultured in osteogenic medium for 16 days. Data are expressed as mean ± standard deviation. An ANOVA followed by Tukey’s test was used to analyze the data. P < 0.005.

Fig 7 shows images of the different markers expressed in osteoblasts. Quantification of the cell markers was carried out after 16 days of osteogenic induction, and cell images were captured at the same time. At this time, the expression of the RANKL protein was found to be increased (relative to the positive and negative markers). In addition, the perinuclear expression of Nanog was higher than that of STRO-1, CD105, and the negative marker CD45RO.

### Confirmation of cell viability of hASC and osteoblasts

**Fig 8:**
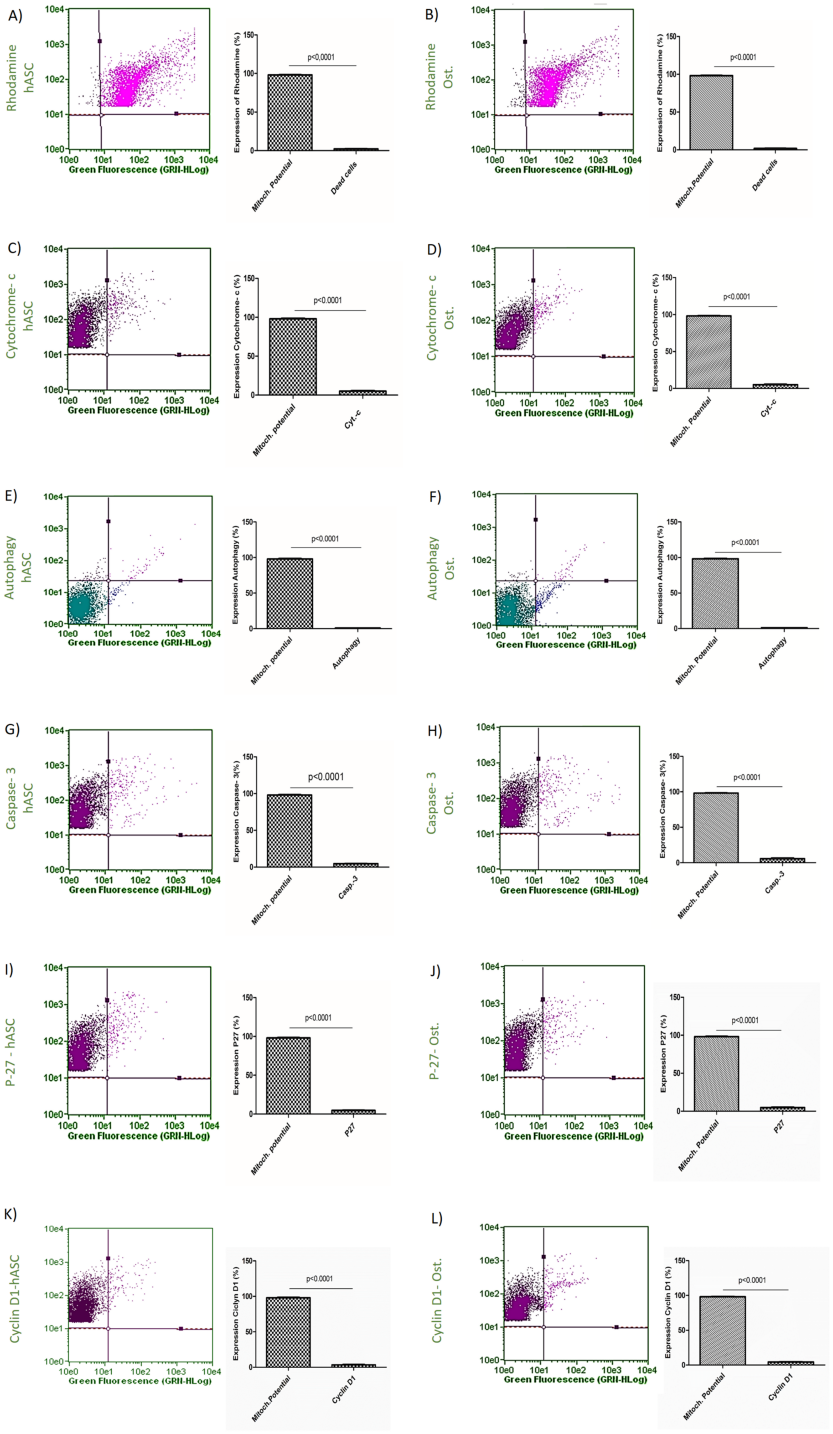
Confirmation of cell viability in hASCs and osteoblasts. (A) High mitochondrial membrane potential in hASCs. (B) High mitochondrial membrane potential in osteoblasts. (C) Cytochrome-c levels in hASCs. (D) Cytochrome-c levels in osteoblasts. (E) Autophagy levels in hASCs. (F) Autophagy levels in osteoblasts. (G) Caspase-3 levels in hASCs. (H) Caspase-3 levels in osteoblasts. (I) P27 levels in hASCs. (J) P27 levels in osteoblasts. (K) Cyclin-D1 levels in hASCs. (L) Cyclin-D1 levels in osteoblasts. Osteoblasts were cultured in osteogenic medium for 16 days and hASCs for the also period of time. The data are expressed as mean ± standard deviation. A *t*-test was used to compare expression levels with P < 0.05 being considered significant.

Our data demonstrated that there was a high mitochondrial membrane potential in both hASCs and osteoblasts (Fig 8A and 8B) and that there was a low expression of all the markers analyzed, cytochrome-c, p62, caspase 3, P27 and cyclin D1 (Fig 8C–8L).

## Discussion

Although some studies report that the osteogenic capacity of hASCs is lower than that of stem cells derived from bone marrow [16,17], hASCs are viable and have good proliferation and differentiation capabilities [18,19]. Additionally, several studies comparing the osteogenic potential of ACCs and stem cells from bone marrow have found that ASCs have greater/better osteogenic potential. [20–22].

hASCs are used in studies since they can be collected easily and a large number of cells can be obtained with rapid expansion of the colonies in in vitro conditions; these factors favor the use of these cells in studies on bone regeneration [23]. However, a series of protocols for the use of hASCs should be established and several aspects, including cell viability, mitochondrial potential, and apoptotic and tumor factors expressed before and after differentiation of hASCs in in vitro conditions, must be evaluated before the clinical application of hASCs [24]. In the present study of osteoblast differentiation from hASCs we showed that the cells had good viability, good stability, and a good proliferation capacity.

Two phases of the differentiation process were followed, the first being the isolation of stem cells, and the second being differentiation into osteoblasts. The detection of vimentin and an examination of positive or negative markers proved these cells were of mesenchymal origin [25]. ASCs are pluripotent cells and exhibit decreased expression of hematopoietic precursors [26,27]. In particular, cytometry tests confirmed the decreased expression of negatives markers, such as CD45RO and CD117, and good expression of the positive markers STRO-1, CD90, CD105, Nanog, and Sox-2. The expression of Sox-2 and Nanog indicates that the cells were pluripotent and undifferentiated [28,29].

Following this phase of isolation, the differentiation process was initiated. As markers of osteoblast differentiation, we examined the expression of RANKL, Osterix, Runx2, Osteopontin, Collagen3A1 and BSP. An increase in the levels of RANKL, cytokine secreted by osteoblasts that actively participates in the bone remodeling process [30], along with alkaline phosphatase and osteocalcin was observed at 16 days of osteogenic induction. Runx2 and Osterix, are transcription factors necessary for bone formation and responsible for controlling the differentiation of mesenchymal stem cells in osteoblasts [31]. In this same period of osteoinduction, the increase of Runx2 and Osterix levels revealed that the cells were differentiated in osteoblast and that these proteins are active so that the progression of bone formation occurs. As well as increased levels of organic matrix proteins such as collagen3A1, BSP and osteopontin can be attributed to the differentiation to mature and functional osteoblasts, confirmed by the formation of a mineralized matrix. Based on these results, our study suggested that from this experimental model, other mediators involved in the remodeling process could be assessed in osteoblasts at 16 days of differentiation from hASCs. Thus, we evaluated the expression of other proposed markers of differentiation by immunofluorescence in osteoblasts after 16 days of osteoinduction. Although Nanog, one of the markers evaluated, is expressed in undifferentiated cells, our data suggest that this protein is present in differentiated cells of mesodermal origin and that other factors, such as epigenetic memory, may have contributed towards the expression of Nanog in osteoblasts. [32]. We also demonstrated the expression of STRO-1, a protein that is expressed at various levels in pre-osteoblasts and mature osteoblasts [33], the expression of CD105, a marker also described in osteoblastic cells derived from human adipose tissue [34], and RANKL, an important stimulator of osteoclastogenesis [30].

Another important parameter that should be observed in the differentiation of stem cells is mitochondrial function [35]. In this study, at 16 days of differentiation, the osteoblasts were found to contain numerous active mitochondria. Thus, we can infer that their respiration rate, as well as their mitochondrial membrane potential, likely contribute to a high level of oxidative metabolism, presumably as a result of the high demand for energy required during the process of hASC differentiation. During osteogenic differentiation, there is an increase in the biosynthesis of enzymes involved in mitochondrial respiration, as this process is important in cell differentiation [36].

A high level of cytochrome-c is related to a reduction in mitochondrial activity and this situation may arise under low amounts of cellular oxygen. This situation can cause the mitochondria to release factors related to apoptosis into the cytoplasm. In this study, the production of cytochrome-c was relatively low in osteoblasts demonstrating that the mitochondrial activity of the cells undergoing differentiation was not compromised.

Caspase-3 is also used as a marker of cell viability, because it participates in the process of cell death, but it may also be involved in the processes of cell proliferation and differentiation [37]. Our data showed that there were low levels of caspase-3 in the osteoblasts, suggesting that the cells were not undergoing active apoptosis, similar to what occurs in normal eukaryotic cells, demonstrating the absence of a microbial infection, as well as adequate cellular homeostasis [38].

The role of autophagy in osteoblasts has also been explored, especially *in vivo*. However, in bone biology, proteins involved in autophagy have been shown to have a significant effect on the process of differentiation and mineralization [39]. Several studies have suggested that intracellular mineralization is mediated by autophagy during the phase of differentiation where secretion of bone matrix occurs [40,41]. However, the role of autophagy in other stages of osteoblast differentiation has not been clearly delineated [42]. In this study, the level of cellular autophagy was low, being similar to the low level of autophagy that occurs in normal cells with normal homeostasis [43]. These results may indicate that the autophagy vacuoles present during stem cell differentiation into osteoblasts are quickly consumed in order to generate energy.

Two other proteins (P27 and cyclin D-1), both of which are related to the control of cell division, were also evaluated in this study. We observed that the production of P27 production in osteoblasts was minimal and we did not observe an increase in the cyclin D1 levels in osteoblasts. These data show that after cellular differentiation the cells are not involved in the inflammatory processes and are not oncogenic [44], two processes that have been suggested to interfere with the process of cellular differentiation [45].

The analysis of the markers studied revealed high hASC activity until and after the differentiation of hASCs into osteoblasts; in both phases, the cells did not exhibit tumorigenic and apoptotic characteristics. These results contribute to progress in bone tissue engineering protocols that aim to promote the viability of cells to cure pathologies involved with bone loss [46]. Since osteogenic cells that differentiate from hASCs do not express factors that compromise cell viability, we suggest that osteogenic differentiation from hASCs may be a useful tool that does not require the use of scaffolds in regenerative medicine. It may aid in the understanding of the bone cytotherapy and bone biofabrication research. Additionally, the present work can be used as an experimental model to understand the expression of different genes expressed in osteoblasts in the presence of hormones, such as triiodothyronine and estrogen, that actively participate in bone metabolism. Understanding how different genes are modulated through these hormones in osteoblasts derived from hASCs may be one more study resource that may aid in research on medicinal interventions to combat bone diseases, such as osteoporosis. With this work, we demonstrated that the established protocol can be applied for basic research, and the results of this study may pave the way for future clinical applications of hASCs.

The proposed methodology used here assesses two different phases of the cell preparation process: the initial isolation of the adipose tissue stem cells and their subsequent differentiation into osteoblasts. Both stages were shown by tests to have high cell viability and a high mitochondrial potential.

The levels of RANKL, Osterix, Runx2, Collagen3A1, Osteopontin and BSP signaled development and osteoblastic maturation from hASC. In addition, osteoblasts showed high cell viability and high mitochondrial potential and absence of tumor characteristics, demonstrating that they are active after cell differentiation. Our results therefore provide a reproducible model for studying the differentiation of hASCs into osteoblasts, which can be used as the basis for future experimental and clinical studies.
